# Beyond silos: An integrated sustainability hierarchy framework

**DOI:** 10.1016/j.isci.2026.116453

**Published:** 2026-06-22

**Authors:** Mengjiao Wang, Fredric Bauer, Joachim Peter Tilsted, Neil Tangri

**Affiliations:** 1 Greenpeace Research Laboratories, BioSciences, University of Exeter, Exeter EX4 4RN, the United Kingdom; 2 Environmental and Energy Systems Studies, Department of Technology and Society, Lund University, 22100 Lund, Sweden; 3 Department of Food and Resource Economics, University of Copenhagen, 1958 Frederiksberg, Denmark; 4 Center for Environmental Public Policy, University of California, Berkeley, Berkeley CA 94720-7320, USA; 5 GAIA, Berkeley, CA 94704, USA

**Keywords:** Environmental science, Environmental management, Environmental resource

## Abstract

To address the gap between global sustainability commitments and action, piecemeal solutions targeting singular impacts fall short. Interventions remain fragmented across sectors and frequently prioritize downstream remediation over upstream prevention. Emphasizing the pressing importance of moving toward more systemic approaches, this paper introduces an integrated sustainability hierarchy framework. Reviewing existing hierarchies on waste, climate change, biodiversity, and more, we provide a cross-domain compass that transcends siloed debates and enables systematic development of policy agendas, impact-oriented financial portfolios, and assessment of interventions. Using the ongoing Global Plastics Treaty negotiations as an illustration, we demonstrate the value of the sustainability hierarchy for multi-dimensional sustainability challenges, going beyond existing domain-specific hierarchies. The framework contributes a practical and theoretically grounded tool for advancing systemic sustainability under conditions of accelerating socio-ecological risk and political backsliding.

## Graphical abstract

**Figure:**
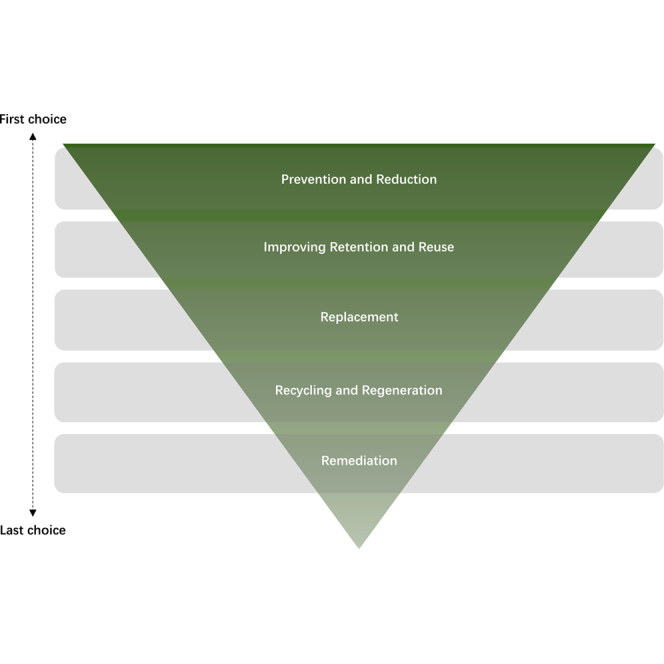

## Introduction

The ongoing United Nations negotiation toward a legally binding instrument to end plastic pollution (hereinafter referred to as the Plastics Treaty) exemplifies a defining tendency of our times: despite increasing awareness of the nature, scale, and urgency of sustainability challenges, and despite public- and private-sector commitments on policy and financing, the interconnected triple planetary crisis of climate change, biodiversity loss, and pollution continues to accelerate. The gap between commitments and action is not only a matter of the number of initiatives launched or the volumes of capital being mobilized but also of the strategic direction these resources push for. In other words, the gap cannot be closed through scaling existing dominant approaches to socio-ecological governance, but it demands a paradigmatic shift in sustainability discourse and decision making.^1^^,^^2^

The planetary boundaries framework has provided a critical diagnostic lens for understanding the scale and systemic nature of the sustainability challenge by identifying a set of Earth system processes that regulate the safe operating space for humanity.^3^ Recent assessments suggest that seven of the nine identified boundaries have already been transgressed, indicating a progressive erosion of Earth system resilience.^4^ The Earth system science literature emphasizes the strong interdependencies among the processes that result in the breaching of planetary boundaries: a set of underlying key socio-economic drivers including fossil fuel use, expansion of industrial agriculture, land use change, and unequal, material overconsumption, which all impact multiple Earth system dynamics.^5^ Despite these insights, responses often remain ad hoc, incremental, and fragmented.^6^^,^^7^ Policy debates, investment strategies, and technological assessments often remain narrowly focused on single pollutants, impacts, products, or sectors rather than on systemic transformations. As a result, resources are frequently and disproportionally directed toward end-of-pipe or end-of-life interventions such as pollution control technologies, waste management systems, and carbon capture infrastructures while squandering critical innovation opportunities that could address root causes of environmental degradation.^8^ Many proposed interventions risk shifting environmental burdens across life cycle stages or impact categories instead.^9^ For example, replacing fossil-based single-use plastics with alternatives from paper or bio-based plastics may alleviate pressures on some environmental indicators while increasing impacts on land use, water consumption, or biodiversity loss.^10^ While debates over individual interventions provide valuable insights, the lack of a systemic perspective risks leading to incoherent governance strategies that constantly fail to meet the long-term sustainability objectives.^11^

As a result of the mismatch between systemic challenges and the piecemeal responses that fail to address the scale and urgency of interlinked ecological degradation, many constituencies consider the term “sustainable development” as a vehicle to perpetuate corporate and institutional interests. Legitimate charges of greenwashing highlight that whilst many actors give the impression of adherence to environmentally sound principles, there are underlying discrepancies between rhetoric, intentions, and action.^12^ Addressing these discrepancies, thus, requires replacing fragmented approaches focusing on piecemeal solutions targeting a singular impact with a comprehensive portfolio approach.

In relation to the Plastics Treaty negotiations, the waste hierarchy has been invoked by some negotiators and scientists as the best available reference for a portfolio approach.^13^ However, despite its merits, relying on the waste hierarchy risks “closing down” the multidimensional sustainability challenge of plastic pollution, reducing it to a waste management issue, instead of “opening up” for an understanding of its broader, systemic nature, encompassing chemical, material, waste, climate, and biodiversity dimensions.^14^ This way of closing down the issue provides justification for countries and lobbyists advocating for a limited scope focused on implementing actions far downstream the impact pathway rather than appreciating alternate visions and pathways that could lead to a transformative change of the whole value chain for sustainability and a just transition.^15^

Given sustainability’s multi-dimensional and evolving nature, there is a strong need for a systematic, cross-domain approach to facilitate consistent sustainability decision making while providing *ex ante* guidance for emerging sustainability challenges that lack dedicated frameworks. Accordingly, this paper introduces the sustainability hierarchy framework (SHF), a framework epitomizing hierarchical thinking, building upon established approaches from different domains. By enabling integrative, coherent, and effective sustainability benchmarking and decision making, the SHF provides decision makers with a framework to systematically evaluate, prioritize, and realign resources across investment portfolios and policy initiatives. Transcending fragmented and siloed responses, the SHF also cultivates opportunities for preventative innovations that could deliver long-term environmental and economic benefits.

The SHF advances a hierarchical portfolio perspective as an organizing lens for sustainability decision making across domains. Consistent with the IMPACT criteria for theory selection,^16^ this perspective is theoretically *interesting*, because it challenges the downstream focus in sustainability governance, which disproportionately allocates resources to waste management, pollution control, and remediation despite evidence that upstream resource use patterns are the dominant drivers of environmental degradation.^17^ The lens *matches* the studied phenomenon as extraction, production, and consumption systems generate interconnected pressures across climate change, biodiversity loss, and pollution, requiring integrated prioritization logics. Simultaneously, it remains *parsimonious* by structuring sustainability interventions into a set of tiers that capture key leverage points along value chains while enabling diversified intervention portfolios. The approach has practical *applicability* as the hierarchy translates into operational prioritization heuristics. The framework demonstrates *conceptual rigor* by clearly defining intervention tiers grounded in system-based analytical principles. Finally, the framework supports empirical *testability*: future research can assess whether portfolios emphasizing higher-tier interventions yield greater system-level improvements than portfolios concentrated on downstream mitigation measures. The hierarchical portfolio perspective, thus, aligns with calls for intervention strategies that target structural drivers and deliver absolute, system-wide reductions in environmental pressures.^18^

## Learning from domain-specific hierarchies

Many existing governance frameworks include hierarchies to guide actions and policies in specific domains. These hierarchies are more nuanced than simple binary categories of advisable and inadvisable actions, such as red/green lists. They acknowledge that less-preferable interventions at the bottom of the hierarchy may be necessary but are to be de-emphasized relative to other, preferable actions. The well-known waste hierarchy,^19^ codified in the European Union (EU) regulations and used globally, ranks waste management interventions from prevention of the quantity and adverse impacts of generated waste to preparing for reuse, recycling, recovery, and disposal as a last resort. However, its prevention tier lacks granular categorization of upstream interventions that could address waste generation at its source, which was disaggregated further by the more recent zero waste hierarchy.^20^ It prioritizes system-level redesign and wasteful consumption avoidance as its highest tier, targeting root causes of linear material flows before waste occurs, although introducing some overlap between its top tiers. The food recovery hierarchy promoted by the United States Environmental Protection Agency^21^ resolves some of this overlap by clearly separating source reduction (e.g., purchasing less) from repurpose/reuse (e.g., feeding people in need), followed by recycling, recovery, and disposal. However, this framework does not address food contaminants introduced throughout the value chain, as its focus remains on food waste prevention rather than broader food system sustainability.

This dimension of toxicity, vaguely reflected in the zero waste hierarchy, is more explicitly addressed in the toxic-free chemical hierarchy of the EU Chemicals Strategy.^22^ This hierarchy places the imperative for materials to be safe and sustainable by design (SSbD) at the top to avoid properties that may be harmful. The lower tiers focus on minimizing and controlling exposure, while finally eliminating substances of concern in waste and secondary raw materials to enable safer recycling and environmental remediation.

In biodiversity and ecosystem conservation, the mitigation hierarchy prioritizes avoidance and minimization before remediation, with compensation as a last resort.^23^ This sequencing is critical to prevent the “biodiversity leak” phenomenon, where researchers found that domestic farmland rewilding in wealthy countries (i.e., tier remediation), without addressing increasing food and wood consumption (i.e., tier avoidance and minimalization), can drive forest destruction in more biodiversity-rich but financially constrained countries.^24^ Acknowledging its value, the mitigation hierarchy is, however, heavily impact-oriented, leading to potential overlap between its top tiers and insufficient prioritization among interventions that affect biodiversity along the value chain.

Hierarchies have also been proposed in the domain of energy and climate. The greenhouse gas management hierarchy, which is based on the experience of sustainability practitioners, distinguishes between absolute elimination of emission sources (e.g., through new business models) and reduction of existing emissions (including efficiency improvement), placing both strategies ahead of substitution and compensation.^25^ The energy hierarchy follows a similar logic with tiers emphasizing demand reduction as a foundation, followed by efficiency measures, before considering alternative sources of energy supply, with offsetting as a final option.^26^ Providing valuable guidance, their primary focus on demand-side interventions, nevertheless, risks failing to translate into tangible changes in the supply system—for instance, the shift toward electrification and renewable energy has not yet led to decreased fossil fuel extraction or supply.

**Figure 1:**
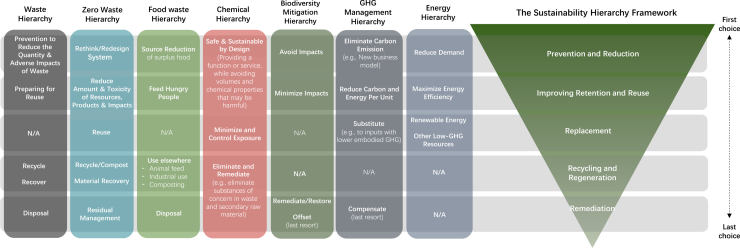
Comparing the proposed generic sustainability hierarchy framework (SHF) with existing hierarchies across domains

While specific interventions and categorizations vary across domains, a shared logic emerges from these frameworks. First, they consistently recognize that interventions differ in systemic effectiveness and therefore, require prioritization within an intervention portfolio rather than being treated as interchangeable options, as summarized in Figure 1. Second, they align on the priority of preventative upstream actions, such as resource use reduction and hazard avoidance, to evade the creation of downstream impacts. This is echoed also in consumer-focused literature, where unsustainable consumption is similarly identified as the root cause for sustainability-related problems.^27^ Third, they position downstream measures, such as treatment, recovery, or remediation, as necessary but inherently less effective interventions, serving to manage residual impacts when higher-tier actions are insufficient.

Building on the strengths and gaps of existing hierarchy frameworks, we find three tensions that motivate a novel, integrated framework.^28^ First, persistent debates on the prioritization of pragmatic downstream approaches over more ambitious and systemic upstream interventions limit the possibility for coherent portfolios. Second, the disconnect between expanding sustainability commitments and deteriorating environmental indicators suggests that current intervention mixes are systematically misallocated with insufficient emphasis on high-leverage interventions. Third, escalating ecological risks and current political backsliding compress the time available for effective action, showcasing the direness of the problem. An integration, reconciliation, and streamlining of these frameworks into a comprehensive, cross-domain, and sector-agnostic framework is, therefore, important to enable consistent decision making across sustainability challenges while retaining clarity, parsimony, and operational relevance.

## Introducing the sustainability hierarchy framework

As a contribution to that integration, we propose the SHF, a strategic, heuristic tool to be applied across domains when planning and evaluating decisions that impact sustainability. The SHF (Figure 1) comprises five hierarchical tiers, drawing on the strongest elements of existing sector-specific hierarchies and informed by a comprehensive understanding of drivers of negative socio-ecological impacts. The SHF aims to be a practical tool for stakeholders and decision makers across sectors at multiple levels.

In descending order, the five hierarchical tiers of the SHF are as follows:

(1) Prevent and reduce resource extraction and consumption: the extraction, processing, and use of fossil fuels and minerals continue to grow, as do overfishing and the conversion of forests to large-scale agricultural production, with significant negative environmental impacts locally and globally. At the highest tier, sustainability decisions should, first and foremost, seek to prevent or minimize the real (absolute) extraction, processing, and consumption of virgin material resources, whether mineral, fossil, or bio-based. This relies on translating science into, for example, global limits for fossil fuel extraction and use, supported by the elimination of harmful subsidies promoting unchecked extraction and production.
(2) Improve retention and reuse of extracted resources: following tier 1, efforts should be made to improve the use of material resources that are already extracted in circular systems through extending the lifespan and value of products as well as their embedded material resources. Such initiatives include enabling and facilitating repairs and modular upgrading, business model change to support reuse and refill, and greater focus on public transportation and shared mobility, thereby maximizing value from already-extracted resources.
(3) Replace problematic and hazardous resources, with inherently safer and more sustainable alternatives (e.g., substitution of hazardous chemicals and switching to renewable energy supplies) and carefully evaluating and avoiding potential risk shifting across generations, geographic, and/or impact categories.
(4) Recycle and regenerate resources, to enable further use of material resources after products’ initial life through measures such as remanufacturing and mechanical recycling, after the top three tiers are rigorously implemented.
(5) Remediate residues and impacts: finally, to address past and remaining damages through measures such as remediation and restoration. While important for environmental and social justice, remediation should complement, not substitute for or maintain priority over higher-tier interventions in the whole sustainability portfolio.

The SHF deliberately excludes offsets and credits, mechanisms sometimes termed “compensation”, as those are not interventions in and of themselves but instead, in practice, substitute lower-tier interventions in one location (such as beach clean-ups, recycling, carbon capture and storage, or tree planting) for responsibilities of higher-tier practices in another location (e.g., to reduce fossil fuel extraction, plastic production, or deforestation). Further, the evidence shows that offsetting schemes have been plagued by systemic problems, which undermine their effectiveness and risk delaying necessary structural and transformative change.^29^^,^^30^ Efficiency improvement is not included as a standalone tier in the SHF either, because it is relevant across all tiers. We see this as consistent with the inclusion of efficiency improvements under varied tiers across existing hierarchies (e.g., in tier design under the chemical hierarchy and in reducing demand, reusing, and recycling under the energy hierarchy).

The SHF tiers can be interpreted as a spectrum of ambition, from addressing the extractivist root at the top to incremental adjustments to established practices at the bottom. Because actions across all five tiers are generally required to achieve sustainability goals, portfolio building and prioritization remain crucial. Upstream prioritization (tier 1 of SHF) is increasingly exemplified in landmark international initiatives and commitments, including the ambition of the Fossil Fuel Non-Proliferation Treaty Initiative, with 18 participating countries supported by 193 subnational governments^31^; of the deep-sea mining moratorium that has already garnered support from 40 countries^32^; and of the 30 × 30 target, to conserve at least 30% of the world’s land, inland water areas, and marine and coastal areas by 2030, enshrined in the Kunming-Montreal Global Biodiversity Framework of the Convention on Biological Diversity,^33^ alongside the concurrent imperative to prevent, phase out, and halt harmful extractive activities. Lower-tier interventions, when well-designed, could benefit specific stakeholders (e.g., site remediation for communities affected by waste colonialism). However, a siloed focus on them alone often externalizes the costs of unsustainable practices, creating new regulatory challenges, while leaving the full scale and scope of socio-ecological crises inadequately addressed. More importantly, they often divert resources and attention away from more transformative, upstream interventions, squandering those innovation opportunities. The SHF is, therefore, designed to enable systematic portfolio building across domains and impact categories for both the private and public sectors, steering aggregate policy support and finance allocation from perpetuating symptom-focused reactive approaches toward transformative interventions and innovations that enable systemic changes.

Effective implementation of the SHF requires a set of guiding principles, building on those in existing hierarchies, including the polluter pays, precautionary, essentiality, human rights, and transparency principles. Implementation must be context-sensitive and involve affected communities, including indigenous peoples, to acknowledge and account for diverse socio-economic conditions and inequalities across scales. Interventions must aim to minimize life cycle externalities and adhere to a “do not export harm” principle across geographical and generational boundaries. A systems perspective of the interlinked planetary challenges is critical to prevent burden shifting, regrettable substitutions, and rebound effects. These considerations intersect with multiple dimensions of justice—distributional, generational, procedural, recognitional, and restorative—and their integration is crucial to fulfill the framework’s potential in advancing a just transition toward sustainability.

The implementation of the SHF in decision-making processes requires procedures for classifying interventions to the different tiers. We propose that interventions should first be classified according to their primary mechanism of impact, i.e., the main pathway for which there is causal evidence of reduced environmental pressure. Second, hybrid interventions combining pathways spanning multiple tiers should, whenever feasible, be disaggregated into components that are assessed and reported separately. If disaggregation is not possible, they should be classified to the lowest tier of their impact mechanism to avoid over-crediting upstream contributions. Third, intervention portfolios that span tiers should, therefore, be represented as distributions showing the share of activity, spending, or expected impact allocated to each tier. An illustration of the operational value of the SHF with suggestions for how to identify interventions in the different tiers is provided in the Global Plastics Treaty discussion in the following sections.

### Limitations of the study

As with any conceptual framework, the SHF carries several limitations that merit reflection. First, while the hierarchy provides a structured prioritization logic, the tiers are generalized to remain broadly applicable across diverse domains, inviting practitioners, researchers, and decision makers to adapt and extend to the specific constraints and opportunities in their context, which influence the viability of different types of interventions. Through the generalized logic, it provides decision makers with a structure for making choices explicit and comparable. Second, classification of interventions involves analytical judgment, particularly for hybrid or system-level measures that span tiers or affect multiple domains. This introduces a risk of inconsistency or even strategic misclassification, emphasizing the need for transparency around assumptions and analytical context. Finally, while the framework aims to mitigate risks for burden-shifting and regrettable substitutions, it must be complemented with empirical tools to assess the magnitude of trade-offs in real outcomes.

## Illustrating the SHF: Guiding the Global Plastics Treaty

**Figure 2:**
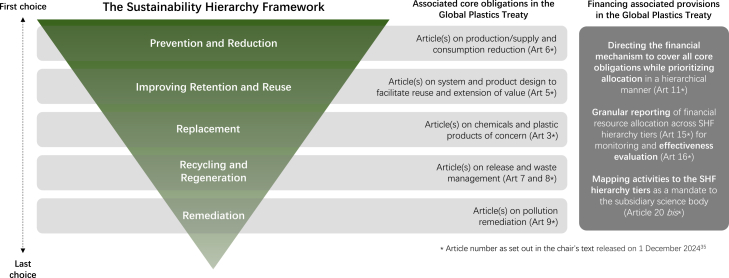
Illustration of the sustainability hierarchy framework used to assess obligations discussed in the Global Plastics Treaty negotiations

The ongoing United Nations negotiation toward the Plastics Treaty provides a timely illustration of the SHF’s value when applied to a sustainability governance domain currently lacking its own hierarchical framework (Figure 2). The absence of a comprehensive framework that could address the multi-dimensional drivers and impacts of plastic pollution throughout the entire life cycle has been limiting negotiators’ ability to articulate, defend, and prioritize transformative upstream solutions. Efforts to use the waste hierarchy as the best available alternative have inadvertently reinforced the misconception that plastic pollution is a waste management issue requiring only waste management and recycling obligations as a response, whereas the mandate to address the full life cycle of plastics necessitates a very different approach. This is a gap that the SHF is uniquely positioned to fill.

Following the SHF, an effective Plastics Treaty should include a comprehensive policy portfolio of strong provisions in order to end plastic pollution and its full spectrum of negative environmental impacts to fulfill the mandate to the Intergovernmental Negotiating Committee (INC) as specified in the United Nations Environment Assembly (UNEA) resolution 5/14.^34^ This portfolio should ideally include at least the core obligations on managing down feedstock extraction and such facilitating subsidies (SHF tier 1), production/supply and consumption reduction (Article 6, as set out in the chair’s text released on December 1, 2024^35^; SHF tiers 1 and 2), system and product design to facilitate reuse and extension of value (Article 5; SHF tier 2), chemicals and plastics of concern (Article 3; SHF tier 3), release and waste management (Articles 7 and 8; SHF tier 4), and environmental remediation (Article 9; SHF tier 5).

Many elements of these key provisions remain under vigorous debate, with higher-tier provisions facing particular threat. Some risk being heavily diluted, for example, a standalone provision on chemicals of concern in the zero draft^36^ has been merged with and listed under Article 3 on plastic products, substantially restricting its scope. Other key provisions have been or are facing total exclusion. For example, language relating to feedstock extraction (SHF tier 1), which was included in the potential options for elements document^37^ drawing upon views expressed by member states, has been entirely removed since the zero draft. Article 6 on the production of primary plastic polymers represents another critical element for effective mitigation of the environmental, climate, and health impact from plastic pollution, supported by robust scientific evidence,^38^^,^^39^^,^^40^ but this is also now at risk in the negotiation. Despite surviving in the zero draft, this article was removed from the chair’s draft text proposals^41^^,^^42^ tabled during a recent round of negotiation (INC5.2 in Geneva, August 2025). This removal was immediately rejected by a majority of countries but was welcomed by a small yet powerful group of countries, which has been advocating for the removal of Article 6 since early negotiation stages. This group, initially identifying as the “Global Coalition for Plastic Sustainability” at INC3, and subsequently as “like-minded countries”, maintains a strong focus on waste management, while arguing against any obligations relating to resource extraction or plastic production. This further exemplifies the limitation of applying only the waste hierarchy, which reinforces the trap of centering negotiations around waste only. The SHF, instead, seeks to address this limitation by providing an alternative and more encompassing framing for the negotiations, guiding its agenda setting and time arrangement in ways that ensure a broader approach to problem definition and enabling exploration of a more holistic portfolio of solutions across all tiers.

The finance negotiations of the Plastics Treaty further emphasize the value of applying the SHF to guide the future financial flows to effectively implement treaty obligations. Technical experts have emphasized that downstream-focused scenarios will increase overall costs and financing complexity compared with comprehensive interventions across the whole life cycle with an emphasis on upstream, higher-tier actions.^43^ Concerns have also been raised over the current financing landscape, with both development and private finance disproportionately directed toward lower-tier waste recycling and recovery,^44^ leaving negligible resources allocated to incentivizing higher-tier innovation and interventions. Nevertheless, proposals singling out or prioritizing waste treatment and pollution remediation received strong support during INC negotiation meetings.^45^^,^^46^ On the other hand, there are countries that see lower-tier interventions as futile without upper-tier interventions to slow the accelerating generation of waste.

The SHF offers a transformative perspective to advance the negotiation by shifting the focus away from a siloed, binary, and confrontational debate that pits one set of interventions (e.g., waste management) against another (e.g., production reduction). Recognizing that the full range of interventions is necessary, the SHF demonstrates that the key to effectively ending plastic pollution requires a comprehensive portfolio approach with strategic prioritization along its hierarchy. This will ensure that higher-tier interventions (e.g., supply reduction) remain an integral part of the Plastics Treaty, supported by appropriate mechanisms to realign, monitor, and evaluate financial flows, both public and private, across all tiers. In practice, this could include, for example, explicit language directing the financial mechanism (Article 11) to cover the full spectrum of above-mentioned obligations while prioritizing the higher tiers as appropriate, as well as eliminating subsidies for production of primary plastic polymers and chemicals of concern.^47^ Complementary provisions on reporting (Article 15) should mandate granular reporting of financial resource allocation cross activities corresponding to SHF tiers for effectiveness evaluation (Article 16), while the guidelines to ensure consistency in mapping activities to the hierarchy tiers could be a mandate to the proposed subsidiary science body (Article 20 *bis*). This would help to address the current official development assistance tracking and evaluation challenges faced by interested organizations like the Organisation for Economic Co-operation and Development (OECD) as well. These treaty obligations on countries will also send a clear policy signal to the private sector to follow suit.

These ongoing negotiations present a critical opportunity to build a comprehensive portfolio reorienting aggregate intervention and financing toward “upstream transformation to a circular plastic economy, which would need further investments in elimination, substitution, and reuse models”, as emphasized by the INC chair. Without this SHF-guided framing change and portfolio-level realignment, the Plastics Treaty risks devolving into a *de facto* waste management and clean-up agreement rather than fulfilling its mandate to truly end plastic pollution—a lost opportunity for the protection of environment, human wellbeing and social justice, as well as for innovation advancement at higher tiers of the value chain. As the negotiations enter their final phase, this strategic orientation and realignment becomes increasingly urgent for ensuring transformative impact.

## Catalyzing systemic change for development and innovation

Today’s environmental urgency, coupled with recent political backsliding on sustainable development, puts future climate and environmental financing in jeopardy, offering a wake-up call to rethink an inefficient system that has long needed re-orientation. Participants at the recent Conference of Parties of the Convention of Biological Diversity similarly noted that “it is not funds we are missing, but rational prioritization,”^48^ pointing to the disproportionate financial spending harmful to biodiversity. Finance and policy, therefore, need to be reimagined not merely for gap-filling but rather as transformative tools for systemic change. In this context, SHF offers a critical compass across domains at all levels.

The SHF contributes a structured basis for improving the design, governance, and evaluation of sustainability portfolios across domains. By prioritizing prevention, reduction, and more efficient resource use, the framework is expected to reduce risks of burden shifting and regrettable substitution. When applied to multi-domain and multi-scalar sustainability challenges, it reorients attention toward upstream obligations, counteracting the tendency to focus interventions on end-of-life management. The framework further enhances transparency and accountability by enabling reporting systems that map interventions, actions, and expenditures to hierarchical tiers, thereby improving the comparability of sustainability claims.

Looking ahead, three avenues of work emerge for decision makers interested in pursuing genuine sustainability by integrating this cross-domain and sector-agnostic SHF approach into:

(1) policy frameworks to send clear political and market signals while creating an enabling environment for transformative changes, which would move beyond narrowly focused binary debates by enabling systematic portfolio building and resource allocation prioritization, thereby ensuring effective implementation, through constructive rather than confrontational dialogues, as evidenced in the aforementioned Plastics Treaty example;
(2) reporting of sustainability data and evaluation of sustainability claims by development of detailed matrix and monitoring systems to track portfolio-level alignment with the SHF; this offers a transformative alternative to the prevailing “box-ticking” mindset and exercise in the current sustainability impact data domain,^49^ reduces reporting burden, and enables better evaluation of aggregate impacts of organizational sustainability efforts; and
(3) strategic decision making on, for example, financing and research and development (R&D) in both the public and private sectors to catalyze advanced and safe upstream innovation, while minimizing the risk of investment into stranded assets and regrettable solutions; by facilitating a transition across the business value creation spectrum from only “do things differently” (e.g., improving recycling and its working conditions) to also “do different things” (e.g., reduced consumption and improved reuse system), this integration also contributes to the two main issues global financial markets endeavor to address: capital not reaching assets that need it and greenwashing.

Implementing the hierarchical thinking embodied in the SHF is no easy task. Significant barriers must be overcome, and tensions between reprioritization and financial logics remain. Despite these limitations, facing the multi-dimensional and ever-evolving sustainability challenges and opportunities, this cross-domain and sector-agnostic SHF provides a point of reference in a comprehensive, constructive, and *ex ante* way to guide the construction and prioritization of sustainability portfolios. The planetary boundaries framework provides a diagnosis of nature’s well-being, which demands urgent transformative changes; the SHF, in complement, provides a much-needed rubric on the prescription to guide policy, intervention, and investment portfolios to enable that change.

## Acknowledgments

The authors thank Ana Paula de Souza, Frankie Orona, Kristian Syberg, David Santillo, and Paul Johnston for their kind suggestions during the preparation of this manuscript.

## Author contributions

Conceptualization, M.W., F.B., J.P.T., and NT; visualization, M.W. and J.P.T.; writing – original draft, M.W., F.B., and J.P.T.; writing – review and editing, M.W., F.B., J.P.T., and N.T.

## Declaration of interests

All authors are members of the Scientists’ Coalition for an Effective Plastics Treaty.
